# Reasoning‐optimised large language models reach near‐expert accuracy on board‐style orthopaedic exams: A multi‐model comparison on 702 multiple‐choice questions

**DOI:** 10.1002/ksa.70222

**Published:** 2025-12-17

**Authors:** Pedro Diniz, Takuji Yokoe, Felix C. Öttl, Hélder Pereira, Rui Henriques, Kristian Samuelsson

**Affiliations:** ^1^ Department of Orthopaedic Surgery Centre Hospitalier Universitaire Brugmann Brussels Belgium; ^2^ Department of Orthopaedic Surgery Hôpital Universitaire de Bruxelles – Hôpital Erasme Brussels Belgium; ^3^ Department of Bioengineering and iBB – Institute for Bioengineering and Biosciences, Instituto Superior Técnico Universidade de Lisboa Lisbon Portugal; ^4^ Division of Orthopaedic Surgery, Department of Medicine of Sensory and Motor Organs, Faculty of Medicine University of Miyazaki Miyazaki Japan; ^5^ Department of Orthopaedic Surgery Balgrist University Hospital Zürich Switzerland; ^6^ Orthopaedic Department Centro Hospitalar Póvoa de Varzim Vila do Conde Portugal; ^7^ Ripoll y De Prado Sports Clinic: FIFA Medical Centre of Excellence Murcia‐Madrid Spain; ^8^ University of Minho ICVS/3B's‐PT Government Associate Laboratory Braga Guimarães Portugal; ^9^ INESC‐ID and Instituto Superior Técnico Universidade de Lisboa Lisboa Portugal; ^10^ Department of Orthopaedics, Institute of Clinical Sciences, Sahlgrenska Academy University of Gothenburg Gothenburg Sweden

**Keywords:** artificial intelligence, clinical decision support, large language models, medical education, orthopaedic surgery

## Abstract

**Purpose:**

The purpose of this study was to compare the accuracy, calibration, reproducibility and operating cost of seven large language models (LLMs)—including four newer models capable of using advanced *reasoning* techniques to analyse complex medical information and generate accurate responses—on text‐only orthopaedic multiple‐choice questions (MCQs) and to quantify gains over GPT‐4.

**Methods:**

From Orthobullets, 702 unique, non‐image MCQs (drawn from AAOS Self‐Assessment Examinations, Self‐Assessment‐Based Questions and Orthopaedic In Training Examination‐Based Questions banks) were extracted. Each question was submitted to the following LLMs: OpenAI o3, Anthropic Claude Sonnet 4, Claude Opus 4 (with/without ‘Extended Thinking’) and Google Gemini 2.5 Pro. Additionally, OpenAI's GPT‐4, GPT‐4o and the open‐weight Gemma 3 27B served as comparators. The primary outcome was overall accuracy. The secondary outcomes were topic and difficulty‐stratified accuracy, calibration (expected calibration error [ECE] and Brier score), reproducibility (flip rate on a retest question subset), latency, token use and cost. Statistical tests included paired McNemar, Cochran *Q*, ordinal logistic regression and Fleiss *κ* (Bonferroni‐adjusted *α* = 0.05).

**Results:**

GPT‐4 achieved 69.7% accuracy (95% CI = 66.2–72.9). All four reasoning‐optimised models scored ≥14 percentage points higher (*p* < 3.3 × 10^−15^); OpenAI o3 led with 93.6% (95% CI = 91.5–95.2), which represents a 34% relative error reduction. Accuracy tended to decline with question difficulty, yet the *reasoning* advantage persisted in every difficulty stratum. Claude Opus 4 showed the best calibration (ECE = 0.023), while GPT‐4 exhibited overconfidence (ECE = 0.215). All models except Gemma 3 27B exhibited non‐zero flip rates. Median query time: 0.9 s (Gemma) to 15.9 s (Gemini 2.5 Pro). Cost: 0 to 29.9 USD per 1000 queries.

**Conclusions:**

Reasoning‐optimised LLMs now answer text‐based orthopaedic exam questions with high accuracy and substantially better confidence calibration than earlier models. However, persistent stochasticity and large latency‐cost disparities may limit clinical deployment.

**Level of Evidence:**

N/A.

**Abbreviations**
95% CI: 95% Wilson confidence interval
AAOS‐SAE: American Academy of Orthopaedic Surgeons Self‐Assessment Examination
CoT: chain‐of‐thought
ECE: expected calibration error
LLMs: large language models
MCQs: multiple‐choice questions
OITE: Orthopaedic In‐Training Examination
USMLE: United States Medical Licensing Examination

## INTRODUCTION

Large language models (LLMs) are artificial intelligence systems that are increasingly used for exam preparation and point‐of‐care information in medicine. In 2023, a general‐purpose LLM, GPT3.5 (OpenAI), surpassed the passing threshold on parts of the United States Medical Licensing Examination (USMLE) [22]. Subsequent iterations—GPT‐4 and GPT‐4o—reached, or exceeded, 80% on USMLE disciplines and clinical skills‐related questions [4, 28, 32]. In the field of orthopaedic surgery, initial studies showed that ChatGPT3.5 answered only 45%–55% of Orthopaedic In‐Training Examination (OITE) items correctly, roughly matching the performance of a first‐year resident [8, 16]. However, GPT‐4 improved to 60%–75% on similar datasets, approaching senior‐resident competence but still failing a full, image‐rich 2019 OITE by scoring 49% [12, 21, 24].

A persistent flaw of early LLMs is that they frequently emitted confident yet erroneous responses [17, 33]. Recently, a series of *reasoning‐optimised* models—which aim to improve accuracy by internally generating step‐by‐step intermediate reasoning—, like OpenAI's o3 (OpenAI), Anthropic's Claude Sonnet/Opus 4 (Anthropic) and Google's Gemini 2.5 Pro (Google LLC), are currently available to the public, making use of the ability to handle long text sequences, multiple internal reasoning passes, and explicit self‐critique to improve answer accuracy, mitigate *hallucinations* (i.e., a model generates text that is not based on actual information in its training data), and, for some models, produce more reliable estimates of its confidence in the answers that were given [1, 7, 29].

Despite these recent technological developments, with a few notable exceptions [11, 13], most current evaluations of LLMs in orthopaedics‐related knowledge remain anchored to GPT‐4 or earlier models [2, 8, 9, 10, 12, 19, 20, 23, 24, 25, 35, 36, 39]. Additionally, studies rarely, if ever, include reproducibility checks [20], calibration metrics [36], or latency‐cost analyses, all of which are crucial for deployment in educational or clinical settings. Furthermore, few head‐to‐head comparisons evaluate multiple *reasoning* models from different providers under identical prompting and temperature conditions [8, 39], making it difficult to discern whether reported gains arise from model architecture, prompt engineering or data set idiosyncrasies.

The present study aims to evaluate seven contemporary LLMs, including four reasoning‐centric models, on a curated corpus of text‐only American Academy of Orthopaedic Surgeons Self‐Assessment Examination (AAOS‐SAE), AAOS‐SAE‐derived and OITE‐derived multiple‐choice questions (MCQs). The primary hypothesis was that each reasoning‐optimised model could improve the reference GPT‐4 accuracy by a minimum of 5 percentage points. Secondary objectives were to quantify topic‐ and difficulty‐specific accuracy, analyse model‐user agreement, evaluate calibration and reproducibility and characterise operational metrics, including latency, token footprint and provider cost.

## MATERIALS AND METHODS

This study compared the performance of seven LLMs on a bank of orthopaedic MCQs. No human participants, patient data or identifiable content were used; therefore, institutional review board approval was not required.

### Question source, composition and selection

Orthopaedic MCQs were manually extracted from the public ‘freetier’ question section of Orthobullets [30] between 7 and 10 June 2025 via its web interface. Three question sources were included: AAOS‐SAE questions (*AAOS‐SAE*, 2007–2013), Self‐Assessment Examination‐based questions (*SBQ*, 2004–2023) and OITE‐based questions (*OBQ*, 2004–2023).

Only text‐based questions were included to allow comparisons with the GPT‐4 model. Questions were sequentially extracted from the Orthobullets website in batches until the estimated number of questions needed was met; thus, no random selection of questions was performed. Details of the sample size calculations for this study are provided in the ‘Statistical Analysis’ section. Questions contained either four or five answer options. To ensure uniqueness, exact duplicates were removed by normalising each stem (lowercasing, punctuation stripping) and hashing, followed by near‐duplicate screening with TFIDF cosine similarity (threshold > 0.80). Only the first unique instance of any duplicate pair was retained, producing the final cleaned dataset. For every question, the stem, the keyed answer, the Orthobullets' platform difficulty label (on a scale from 1 to 5, corresponding to *easy* to *hard*), the proportion of users selecting each option, and one of twelve major orthopaedic topics, following the categories proposed by Orthobullets, were recorded.

### LLM tested

Seven publicly available LLMs released between March and May 2025 were evaluated. Four are considered *reasoning* models: OpenAI o3 (version: ‘o3‐2025‐04‐16’), Anthropic Claude Sonnet 4 (version: ‘claude‐sonnet‐4‐20250514’), Claude Opus 4 (version: ‘claude‐opus‐4‐20250514’, with and without ‘Extended Thinking’), and Google Gemini 2.5 Pro (version: ‘gemini‐2.5‐pro‐preview‐06‐05’).

In the literature, Chain of thought (CoT) prompting refers to asking a model to ‘think step by step’, which aims to improve response accuracy by explicitly stating the model's intermediate reasoning processes [38]. Experimental evidence shows that CoT prompting improves model performance on arithmetic tasks (up to 40%), common‐sense tasks (up to 10%) and symbolic tasks (up to 90%) [38].

Reasoning‐optimised LLMs internally generate and refine multi‐step reasoning paths—often through self‐critique or additional hidden CoT‐like *thinking* passes—to improve the accuracy and reliability of their final answers. For example, the ‘Extended Thinking’ option in Anthropic's Opus 4 grants the model an auxiliary scratch‐pad of hidden tokens for internal reasoning passes before it returns the user‐visible answer, similarly to an implicit CoT. In this study, we did not provide external CoT exemplars or few‐shot demonstrations; instead, we relied on the models' native behaviour or optional settings to produce intermediate reasoning.

In addition to the four reasoning‐centric LLMs, we evaluated GPT‐4o (version: ‘gpt‐4o‐2024‐08‐06’), which is the successor to GPT‐4 and not tuned explicitly for CoT reasoning, and Gemma 3 27B (version: ‘gemma‐3‐27b‐it’; Google LLC), a novel open‐weights transformer, to give context for how proprietary mainstream and current open‐source models fare against both GPT‐4 and the reasoning‐optimised group. All queries were executed via the LLM providers' application programming interfaces (APIs) using the model versions current as of 17 June 2025.

### Prompting protocol

Each question was submitted as an isolated API call. All models received the same system prompt and user prompt template. The system prompt and all remaining parameters used in the study are summarised in Table 1. The temperature parameter was fixed at 0.0 for all models. Because OpenAI's o3 endpoint does not allow a temperature parameter, a fixed seed was supplied instead to promote determinism. Currently, the Anthropic and Google APIs do not expose a seed parameter. The Anthropic API required that a maximum number of tokens per answer be specified, which was set at 1024 tokens to ensure that a single‐letter answer would be returned while allowing enough tokens for the models' CoT process. No maximum token limit was set for the remaining models. No CoT exemplars or few‐shot examples were externally supplied. Wall‐clock latency, prompt tokens, completion tokens and provider‐reported cost were recorded for every request where such metadata was available.

**Table 1:** Model and prompting parameters.

**Base prompt**				
You are an orthopaedic surgeon undergoing a certification examination Your goal is to answer the following question as precisely as possible You work in the United States. You are being subjected to an exam involving multiple‐choice questions. Only one option is correct. You need to select the option you deem the most likely to be the correct answer. You need to carefully evaluate each option and then make a decision about which one is the correct answer. If you are unsure, pick one anyway. If you fail your exam, you will be barred from working Your goal is to pick the correct answer In your output, provide *only* the letter corresponding to the correct choice and nothing more The question:{question}				
**Confidence assessment prompt**				
On a scale from 0 to 1, how confident are you that option {answer} is correct? Respond with a single decimal number (e.g., 0.79). Do not include the letter or any other text.				
**Model parameters**				
*Model*	*Provider/Version*	*Temperature*	*Seed*	*Other fixed params*
GPT‐4	OpenAI, ‘gpt‐4‐0613’	0.0	42	—
GPT‐4o	OpenAI, ‘gpt‐4o‐2024‐08‐06’	0.0	42	—
o3	OpenAI, ‘o3‐2025‐04‐16’	N/A^+^	42	—
Claude Sonnet 4	Anthropic, ‘claude‐sonnet‐4‐20250514’	0.0	N/A	max_tokens = 1024
Claude Opus 4	Anthropic, ‘claude‐opus‐4‐20250514’	0.0	N/A	max_tokens = 1024
Claude Opus 4 (ET)	Anthropic, ‘claude‐opus‐4‐20250514’	1.0^a^	N/A	budget_tokens = 1024 max_tokens = 2024
Gemini 2.5 Pro	Google, ‘gemini‐2.5‐pro‐preview‐06‐05’	0.0	N/A	—
Gemma 3 27B	Google, ‘gemma‐3‐27b‐it’	0.0	N/A	—

*Note*: N/A^+^: the o3 model does not allow setting the temperature parameter.

Abbreviations: ET, extended thinking.

^a^ Temperature must be set to 1.0 when using the ET option.

### Outcomes

The primary outcome was overall accuracy, defined as the proportion of questions answered correctly by each model. Secondary outcomes comprised accuracy stratified by topic and difficulty level; pair‐wise and overall model agreement; whether the models tended to choose the same answers as the users when an incorrect option was chosen; whether models tended to succeed on the same questions that users people tended to answer correctly; calibration assessment; reproducibility of the models' answers; and latency, token usage and USD cost per 1000 queries.

### Calibration assessment

Calibration assessment was performed by querying the models with an additional ‘user’ prompt, asking how confident they were regarding their answer, while injecting the question and the model's answer as ‘user’ and ‘assistant’ content, respectively. The models were asked to return only their confidence in their previous answer, as rated between 0.00 and 1.00 in a two‐decimal format, with 1.00 denoting 100% confidence [33]. The decision to assess model calibration via prompting was made to ensure a similar assessment method across models, as not all models allowed the calculation of token probability via the log probabilities parameter in the API.

### Reproducibility assessment

Determinism was assessed by re‐querying a random sample of questions twice for each model, with no significant delay (i.e., immediately afterwards). The flip rate (percentage of answers that changed between repetitions) and its 95% Wilson confidence interval (95% CI) were calculated. A model was classified as deterministic if it maintained the same answers between runs (a flip rate of zero).

### Statistical analysis

Descriptive statistics use the mean and standard deviation when data are normally distributed; otherwise, the median and interquartile range are used. Normality was assessed using the Shapiro–Wilk test. The 95% CIs were calculated using the Wilson score method.

Accuracy for each model was summarised with 95% CIs. Pair‐wise comparisons of each reasoning‐based model versus GPT‐4 were performed using exact McNemar tests with a Bonferroni adjustment. Overall differences across all seven models were assessed with a Friedman test. Topic‐ and difficulty‐specific effects were examined with Cochran's *Q*, and the relationship between performance and difficulty was explored with ordinal logistic regression.

Agreement between models was quantified with Cohen's *κ* (pair‐wise) and Fleiss *κ* (overall). To assess alignment with users, we compared the distribution of incorrect model choices across user‐consensus strata (high/mid/low) using chi‐square tests, and correlated item‐level model correctness with the proportion of users answering correctly using Spearman's *ρ*.

Calibration was derived from prompted confidence and summarised with expected calibration error (ECE), Brier score and five‐bin reliability curves. Reproducibility (determinism) was evaluated on repeated queries as the flip rate. All tests were two‐sided with *α* = 0.05 (before correction unless stated).

### Sample size estimation

For McNemar comparisons, 662 items provide 80% power to detect a 5‐percentage‐point absolute difference (baseline 65%, within‐item correlation *r* = 0.55; two‐sided *α* = 0.05). Therefore, 700 questions were targeted. Calibration precision was assessed using 1000 bootstrap resamples, aiming for a 95% CI half‐width of ≤0.02 on ECE; simulations indicated that approximately 400 questions, stratified by topic and difficulty, were sufficient. To estimate flip rates with a 95% CI width ≤5%, at least 75 items per model were re‐queried.

## RESULTS

**Figure 1:**
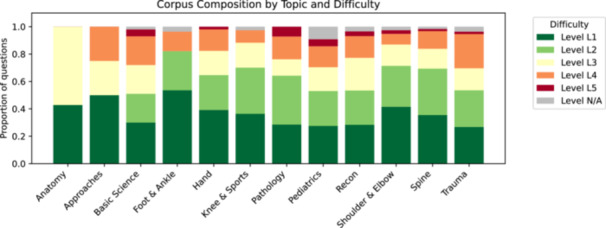
Question corpus composition. Bars are normalised to 100% so that segment heights reflect the fraction of questions in each difficulty category. The full corpus comprises 702 multiple‐choice items drawn from AAOS‐SAE, SBQ and OBQ sources.

The question bank comprised 702 unique, text‐only MCQs spanning the twelve Orthobullets categories and five predefined difficulty strata (Figure 1). Forty‐four per cent of questions originated from the AAOS‐SAE series, 29% from SBQ and 27% from OBQ.

### Primary outcome

**Figure 2:**
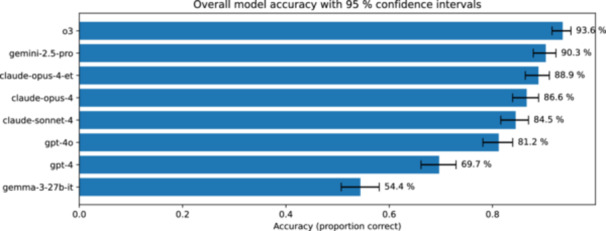
Overall model accuracy with 95% confidence intervals. Horizontal bars show the proportion of correct answers for each large language model, sorted from highest to lowest accuracy. Error bars represent 95% confidence intervals calculated using the Wilson score method.

Reasoning‐based models greatly surpassed GPT‐4 in answering board‐style MCQs, indicating a clear step‐up in practical test‐taking performance. Across the question corpus, GPT‐4 answered 69.7% of questions correctly (95% CI = 66.2–72.9) and served as the reference comparator. All four *reasoning* models surpassed GPT‐4 by more than 14 percentage points (statistical significance confirmed by exact paired McNemar testing, Bonferroni‐adjusted *p* < 3.3 × 10^−15^ for every comparison), with OpenAI o3 achieving the highest score (93.6%; 95% CI = 91.5–95.2) (Figure 2). A global Friedman test confirmed substantial between‐model heterogeneity (*χ* ^2^ = 778.9, *p* < 6.7 × 10^−16^).

### Secondary outcomes

#### Topic‐ and difficulty‐specific performance

**Figure 3:**
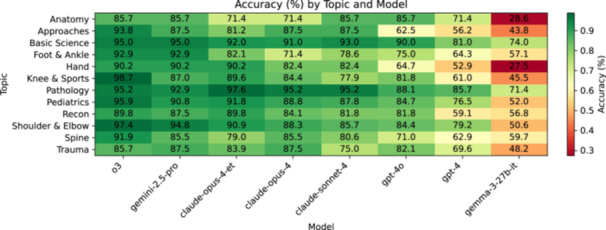
Model accuracy by topic (heat map). Each cell shows the percentage of correctly answered questions for one large language model (columns, ordered left to right by descending overall accuracy) on each topic (rows, number of questions per topic varies). Values are annotated inside each cell. Colour intensity corresponds to accuracy from 30% (*lightest*) to 100% (*darkest*), as indicated by the colour bar. All percentages are based on the complete 702‐question corpus.

**Figure 4:**
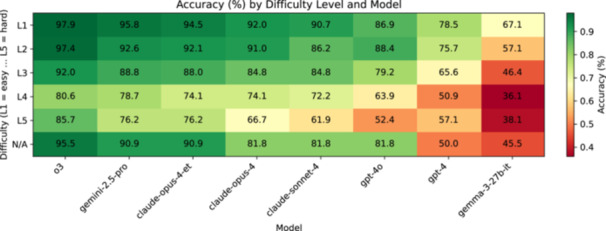
Model accuracy by difficulty level (heat map). Each row represents one Orthobullets difficulty tier (L1 = easiest, L5 = hardest, N/A = uncategorised) and each column one large language model (ordered left to right by overall accuracy). Cells contain the per cent‐correct score (annotated) for that model at that difficulty; shading follows the colour bar scale from 40% (*light*) to 100% (*dark*). All values are based on the full 702‐question corpus (the number of questions per tier varies).

The advantage of reasoning‐based models persisted across subspecialties and even at higher difficulty tiers, suggesting robust performance gains. The heat‐map revealed consistent model rankings across the 12 topics (Figure 3), although absolute accuracy varied (Cochran's *Q*, *p* < 0.05 for all models). Accuracy tended to decline with rising question difficulty levels (Figure 4). Ordinal‐logistic regression revealed independent main effects of both model and difficulty, yet weak interaction, suggesting that reasoning‐optimised models maintained their advantage even on the most challenging questions. For example, o3 dropped from approximately 97.9% on Level 1 to approximately 85.7% on Level 5 questions, yet still outperformed GPT‐4 by more than 20–30 percentage points at every tier.

### Agreement analyses

The agreement between models varied from weak to moderate. Pair‐wise Cohen *κ* ranged from 0.54 (Claude Sonnet 4 vs. Claude Opus 4 with ‘Extended Thinking’) to 0.07 (Gemma 3 27B vs. o3), while overall seven‐rater agreement was moderate (Fleiss *κ* = 0.28).

Models tended to pick the same question distractors as human users when incorrect answers were chosen. Across all wrong answers, 61% corresponded to the distractor most popular with users, 19% to a mid‐rank choice and 20% to a rarely selected option; this distribution did not differ between models (*χ* ^2^ = 31.4, *p* = 0.067).

Model performance showed only modest correlation with user accuracy patterns. Spearman's *ρ* ranged from 0.24 to 0.33 across the seven models, showing a loose‐to‐moderate rank alignment with Orthobullets' user base accuracy. Notably, o3—despite having the highest absolute accuracy—showed the lowest correlation (*ρ* = 0.24), suggesting that its advantage is centred on questions that users find most challenging. Values for agreement between each model and GPT‐4, as well as with Orthobullet's users, are presented in Table 2.

**Table 2:** Agreement with GPT‐4 (using Cohen's *κ* for pair‐wise agreement between models) and Orthobullets’ users (using Spearman's rank‐correlation coefficient), calibration assessment and flip rate. Calibration and flip rates were assessed on 400‐ and 75‐question subsets, respectively.

Model	Agreement		ECE (95% CI)	Brier score (95% CI)	Flip rate (%)
	GPT‐4	Users			
o3	0.15	0.24	0.181 (0.160–0.200)	0.083 (0.072–0.095)	5.6
Gemini 2.5 Pro	0.23	0.29	0.054 (0.029–0.080)	0.081 (0.059–0.105)	2.8
Claude Opus 4 (ET)	0.31	0.29	0.040 (0.020–0.065)	0.094 (0.073–0.117)	2.7
Claude Opus 4	0.34	0.27	0.023 (0.006–0.049)	0.115 (0.093–0.139)	1.4
Claude Sonnet 4	0.35	0.3	0.044 (0.018–0.082)	0.132 (0.108–0.158)	1.4
GPT‐4o	0.42	0.33	0.062 (0.033–0.096)	0.143 (0.117–0.169)	1.4
GPT‐4	N/A	0.33	0.214 (0.173–0.257)	0.281 (0.242–0.320)	2.8
Gemma 3 27B	0.39	0.31	0.348 (0.300–0.397)	0.358 (0.320–0.397)	0.0

Abbreviations: CI, confidence interval; ECE, expected calibration error; ET, extended thinking.

### Calibration assessment

On the 400‐question calibration sample, Claude Opus 4 achieved the highest reliability (ECE = 0.023, 95% CI = 0.006–0.049), o3 combined top accuracy with moderate mis‐calibration (ECE = 0.181, 95% CI = 0.160–0.200), whereas GPT‐4 was markedly over‐confident (ECE = 0.215, 95% CI = 0.173–0.257) (Table 2). Improved calibration indicates that model confidence closely tracks correctness, a desirable feature for safe educational feedback.

### Reproducibility

Although models mainly produced identical answers when asked the same question twice, repeated querying of 75 randomly selected questions demonstrated that, except for Gemma 3 27B, none of the models were truly deterministic. The flip rates for each model are listed in Table 2.

### Efficiency metrics

**Figure 5:**
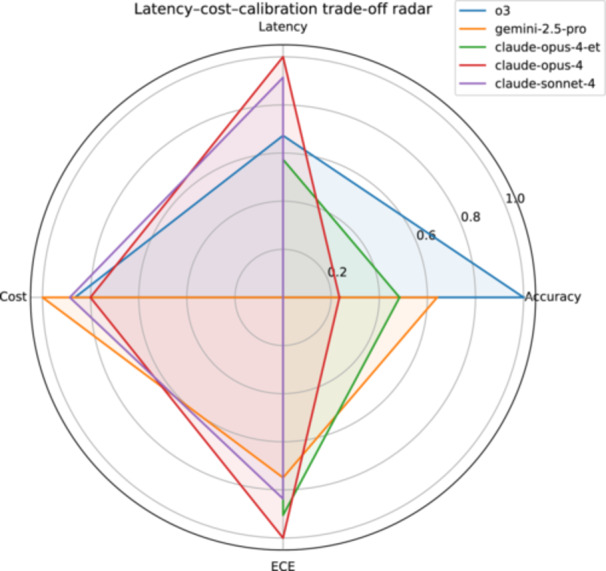
Latency‐cost trade‐off radar. Each polygon represents one model (legend, ordered by descending overall accuracy) across four min‐max normalised metrics: proportion correct (Accuracy), inverse median response time in seconds (Latency*), inverse provider cost per 1000 queries in USD (Cost*), and inverse expected calibration error (ECE*). All metrics were scaled to 0–1, so larger radial values denote better performance on every axis. Data are based on the complete 702 questions per model for latency and cost, and on 400 questions for ECE. *: values were inverted before scaling so that lower values yield higher scores.

Reasoning models achieved markedly higher accuracy at the expense of longer response times and higher per‐query costs, with Claude Opus 4 (Extended Thinking) being the slowest and most expensive option, and GPT‐4o and Gemma 3 27B providing the fastest and lowest‐cost outputs. A summary of variation across models regarding latency, token usage and USD cost per 1000 queries is presented in Table 3. A radar plot comparing these metrics is shown in Figure 5.

**Table 3:** Efficiency metrics. Costs use provider pricing current on 24 June 2025.

Model	Latency (s), median [IQR]	Input tokens, median [IQR]	Output tokens, median [IQR]	Cost (USD/1000 Q)
o3	5.02 [3.48–8.80]	223.00 [204.25–249.00]	275.00 [147.00–531.00]	4.2878
Gemini 2.5 Pro	3.68 [3.07–4.88]	252.00 [232.00–284.75]	4.00 [4.00–4.00]	6.1849
Claude Opus 4 (ET)	10.02 [8.66–11.35]	280.00 [260.00–312.75]	334.00 [291.00–382.00]	29.9041
Claude Opus 4	6.82 [2.05–8.29]	252.00 [232.00–284.75]	242.00 [4.00–308.00]	3.6519
Claude Sonnet 4	15.93 [13.72–19.11]	218.00 [200.00–245.00]	1.00 [1.00–1.00]	0.2943
GPT‐4o	0.82 [0.72–1.02]	224.00 [205.25–250.00]	1.00 [1.00–1.00]	0.6009
GPT‐4	1.49 [1.16–1.79]	225.00 [207.00–252.00]	8.00 [5.00–11.00]	2.8697
Gemma 3 27B	0.87 [0.76–0.97]	222.00 [204.00–249.00]	1.00 [1.00–1.00]	0.0000

Abbreviations: ET, extended thinking; IQR, interquartile range.

## DISCUSSION

Reasoning‐based large language models substantially outperformed GPT‐4 in answering board‐style orthopaedic questions. In this comparative evaluation of systems available in mid‐2025, all reasoning models exceeded GPT‐4 by wide margins, surpassing the pre‐registered superiority threshold and confirming the primary hypothesis.

The absolute magnitude of o3's gain—93.6% versus GPT‐4's 69.7%—represents a 34% relative error reduction and exceeds the highest score in the 2024 OITE (218 correct questions out of 254, or 86%) [14]. Although the question corpus in this work excluded imaging‐based questions, the magnitude of improvement suggests that contemporary reasoning‐centric architectures can now solve the textual component of orthopaedic board exams at, or beyond, expert level.

Performance gains were heterogeneous but widely distributed across the question corpus. Notably, o3 and Gemini 2.5 Pro achieved ≥90% accuracy in 10 of the 12 topics, including Paediatrics—a domain traditionally underrepresented in pretraining corpora [27]. Scores in the Anatomy and Trauma topics tended to be on the lower end across models, which may be due to the underrepresentation of Anatomy‐ and Trauma‐specific content in the pretraining corpora. The relatively larger proportion of Level 4 difficulty questions in the Trauma topic may also partially explain why models tended to underperform in this topic, compared to, for example, Knee & Sports, which contained a much larger proportion of questions with difficulty Levels 1 to 3.

Accuracy in the question corpus tended to decline from Level 1 to Level 5 difficulty grading. Apple's June 2025 *Illusion of Thinking* study observed that reasoning‐centric LLMs, or Large Reasoning Models as they are referred to in the aforementioned study, perform well only up to a compositional‐complexity threshold and then enter a ‘collapse’ regime in which answer accuracy drops sharply despite generous token budgets and explicit CoT prompts [31]. The Levels 4 and 5 orthopaedic questions in the corpus, which may entail multi‐step treatment algorithms, appear to elicit a similar effect, reinforcing the view that reasoning‐centric LLMs still lean on pattern heuristics rather than robust causal chains. Importantly, ordinal logistic analysis revealed that the associative strength between the model's behaviour and difficulty is not statistically significant (*p* = 0.41), corroborating the observed stratified accuracy differences of over 20 percentage points on the most challenging questions between the *reasoning* models against GPT‐4 and open‐weight baselines.

Spearman correlations (*ρ* = 0.24–0.33) indicate only modest alignment between user difficulty and model success; notably, o3 excelled on questions that users most often miss. This divergence implies that these LLMs could function as ‘knowledge‐gap detectors’ and function as *complementary intelligence*, provided outputs are triaged by a confidence‐gating or cascaded‐ensemble workflow [3, 18]. Because query time and cost vary by more than an order of magnitude across models, real‐world deployments will need tiered routing that pairs inexpensive, fast models with high‐fidelity ones for critical cases [6].

The quality of calibration varied considerably. Claude Opus 4 achieved an ECE of 0.023—far below GPT‐4 (0.214). The need for well‐calibrated confidence in LLMs aligns with new governance regimes: the European Union's AI Act classifies any system that can influence clinical decision‐making as *high‐risk* and mandates transparent uncertainty disclosures [5], while the Food and Drug Administration's 2025 draft guidance for AI‐enabled device software states that ‘clinical decision support devices may need longitudinal data with survival analysis, calibration analysis and/or discrimination analysis’ [34]. *Reasoning* LLMs that combine high accuracy with low ECE, therefore, are closer to clearing a key regulatory hurdle. Notwithstanding, all models except Gemma 3 27B exhibited non‐zero flip rates on repeat queries, which underscores residual stochasticity despite attempts to increase model determinism. Such variability makes audit trails more complex and collides with the traceability requirements in ISO/IEC 42001:2023 [15]. Pairing temperature‐zero decoding with fixed seeds and majority‐vote self‐consistency is a possible mitigation route [26, 37].

The obtained results show that LLMs are becoming real‐world educational and clinical aids, which brings a new set of responsibilities and pitfalls. Automation bias—over‐trusting a confident but incorrect answer—remains a risk, as the calibration results in the present study show that even the best‐performing models still err at times while exhibiting miscalibrated confidence in their answers. Integrating clear confidence cues in the model's response, along with training users to question uncertain outputs and build AI literacy, alongside traditional evidence‐based reasoning, is needed. Because latency and cost differ by an order of magnitude across models, practical deployments may need to adopt a tiered routing approach: dispatch rapid, low‐cost models for routine queries and reserve slower, high‐fidelity models for more complex queries. With these safeguards, *reasoning* LLMs can evolve from supplemental revision aids to first‐line instructional resources—provided human oversight stays firmly in the loop, at least for the moment.

From an educational standpoint, reasoning‐based LLMs capable of consistently answering board‐style questions with high accuracy could serve as reliable tools for self‐assessment and guided study. Their improved calibration allows users to gauge not only whether an answer is correct, but also how confident the system is—helping trainees identify areas of uncertainty. In clinical training contexts, such systems may also support just‐in‐time learning by providing structured reasoning for diagnostic and management choices. However, their use should remain supervised, as over‐reliance on confident but incorrect answers could reinforce misconceptions.

### Limitations

This study presents a few limitations. First, potential data leakage cannot be ruled out; portions of the Orthobullets question corpus may exist in the models' pretraining data. The overlap between user and model error patterns suggests that strict answer memorisation is not at play, but does not eliminate the risk. Other factors may also have positively biased accuracy, including the use of MCQ items that provide structured cues and familiar phrasing, which may mirror examples present in publicly available medical content used for model training, as well as question stems that resemble guideline‐based statements. Second, the exclusion of image‐based questions narrows external validity—radiograph and CT interpretation are central to orthopaedics. Third, topic imbalance, which arose from relying on the Orthobullets' ‘free tier’ questions, may skew topic‐level comparisons. Fourth, the MCQ format supplies cues absent in free‐text clinical reasoning and could inflate model performance. Fifth, cost and latency estimates are snapshots and may shift rapidly. Finally, the technological landscape surrounding LLMs is changing rapidly; therefore, the models tested in this study may become outdated and surpassed by newer generations.

## CONCLUSION

Reasoning‐centric LLMs now solve text‐based orthopaedic exam‐style questions at a much higher level of performance compared to GPT‐4, with OpenAI's o3 model reaching 93.6% accuracy. Claude Opus 4's low ECE (0.023) indicates that reliable confidence estimates are emerging; however, residual flip rates, uncertain imaging analysis capabilities and wide latency‐cost spreads remain important challenges for clinical deployment.

## AUTHOR CONTRIBUTIONS

Pedro Diniz conceptualised the study, performed data extraction, prepared the computer scripts to run the experiment, analysed the results and drafted the manuscript with feedback from Takuji Yokoe, Felix C. Öttl and Rui Henriques. Hélder Pereira and Kristian Samuelsson revised the manuscript. All authors have read and approved the final submitted manuscript.

## CONFLICT OF INTEREST STATEMENT

KS is a member of the Board of Directors of Getinge AB (publ) and medtech advisor to Carl Bennet AB.

## ETHICS STATEMENT

The ethics statement is not available.

## Data Availability

Data and code can be provided upon reasonable request.
